# Voluntary wheel running promotes lymphangiogenesis in slow-twitch muscle in young mice

**DOI:** 10.3389/fphys.2025.1654445

**Published:** 2025-10-10

**Authors:** Yuma Tamura, Takafumi Kawashima, Aoi Kodama, Rui-Cheng Ji, Yuta Itoh, Nobuhide Agata, Keisuke Kawakami

**Affiliations:** ^1^ Physical Therapy Research Field, Graduate School of Medicine, Oita University, Yufu, Japan; ^2^ Department of Rehabilitation, Akeno-Central Hospital, Oita, Japan; ^3^ Faculty of Welfare and Health Science, Oita University, Oita, Japan; ^4^ Graduate School of Welfare and Health Science, Oita University, Oita, Japan; ^5^ Faculty of Rehabilitation Science, Nagoya Gakuin University, Nagoya, Japan; ^6^ Faculty of Health and Medical Sciences, Tokoha University, Hamamatsu, Japan

**Keywords:** skeletal muscle, lymphatic vessels, blood capillary, voluntary wheel running, aged mice

## Abstract

**Introduction:**

Lymphatic vessels contribute to tissue homeostasis. Although the lymphatic vessels in skeletal muscle are known to undergo structural changes under certain conditions, such as atrophy and injury, effects of exercise on intramuscular lymphatic vessels remain unclear.

**Methods:**

This study was aimed at investigating whether 8 weeks of voluntary wheel running (VWR) induces histological changes in lymphatic and blood capillaries, and whether these responses are related to age and myofiber type. Young (3-month-old) and aged (18-month-old) male C57BL/6 mice were assigned to sedentary or VWR groups. The soleus (SOL; slow-twitch) and plantaris (PLAN; fast-twitch) muscles were analyzed using immunohistochemistry and quantitative polymerase chain reaction.

**Results:**

In young mice, VWR increased the quantity of type I myofibers and significantly enhanced the density of lymphatic vessels and blood capillaries in the SOL, besides upregulating the expression of vascular endothelial growth factors, VEGF-C and VEGF-D. These changes were not observed in aged mice or in the PLAN of mice in either age group.

**Discussion:**

Although aged mice showed a similar increase in the quantity of type I myofibers, they did not exhibit corresponding vascular remodeling, which suggests that aging reduces responsiveness to exercise-induced angiogenic and lymphangiogenic signals. Overall, these findings indicate that VWR promotes lymphangiogenesis and angiogenesis in slow-twitch muscle in young mice, probably as an adaptive response to meet the increased oxygen demand. Exercise-induced vascular and lymphatic remodeling in skeletal muscle is significantly influenced by age and myofiber type, highlighting a reduced adaptive capacity of aged muscle that may impact strategies for promoting vascular health through physical activity.

## 1 Introduction

Lymphatic vessels are an integral component of the circulatory system, playing essential roles in lipid and immune cell transport as well as in the regulation of inflammatory response (Angeli and Lim, 2023; Hu et al., 2024). In peripheral tissues, lymphatic vessels are also responsible for collecting interstitial fluid that leaks from blood capillaries, and thereby contribute to fluid homeostasis (Oliver et al., 2020). Lymphatic vessels are also present within skeletal muscles. Kivelä et al. (2007a) were the first to report that small lymphatic vessels are located adjacent to blood capillaries between myofibers in human and murine skeletal muscles.

The distribution and morphology of intramuscular lymphatic vessels are altered under pathological conditions, such as muscle atrophy and injury (Kawashima et al., 2021; Tamura et al., 2024). Furthermore, recent report suggested that aging and myofiber type also influence the structural characteristics and distribution of lymphatic vessels (Taketa et al., 2024). These findings indicate that the architecture of lymphatic vessels within skeletal muscles is affected not only by pathological factors but also by physiological factors.

Changes in lymphatic vessels are primarily mediated by the expression of lymphangiogenic factors, such as vascular endothelial growth factors, VEGF-C and VEGF-D, and their receptors (VEGFR-3) (Tammela and Alitalo, 2010; Ji, 2012; Suarez et al., 2023). These molecules regulate the distribution and morphology of intramuscular lymphatic vessels in response to pathological and physiological stimuli (Ji, 2018; Kawashima et al., 2021; Pu et al., 2021; Tamura et al., 2024) and are critical for maintaining homeostasis in skeletal muscles.

The effects of exercise on intramuscular lymphatic vessels remain largely unclear. Although studies using treadmill-based exercise were previously conducted, no significant changes or decrease in intramuscular lymphatic vessels were observed after such interventions (Kivelä et al., 2007b; Greiwe et al., 2016). Voluntary wheel running (VWR) has recently gained attention as an exercise intervention that closely resembles natural locomotor patterns and does not disrupt the normal nocturnal circadian rhythm of animals (De Bono et al., 2006; Goh and Ladiges, 2015; Manzanares et al., 2018). VWR improves muscular endurance and enhances metabolic function in young mice (Pellegrino et al., 2005; LaBarge et al., 2016; Hedges et al., 2019). Furthermore, VWR prevents age-related decline in muscle function in aged mice (Yoshioka et al., 2019; Baek et al., 2022), underscoring its potential impact on skeletal muscles. VWR also increases blood capillary density and upregulates angiogenic factors such as VEGF-A in both slow- and fast-twitch skeletal muscles (Waters et al., 2004; Jackson et al., 2015; Hyatt et al., 2019; Zmudzka et al., 2025). However, the effects of VWR on intramuscular lymphatic vessels have not been examined. Investigating changes in intramuscular lymphatic vessels induced low-intensity and low-stress exercise like VWR may provide clues useful for understanding lymphedema rehabilitation and age-related muscle function decline.

Therefore, in this study, we investigated the histological changes in intramuscular lymphatic vessels and blood capillaries in young and aged mice subjected to VWR and evaluated the effects on the levels of lymphangiogenic and angiogenic factors in these animals.

## 2 Materials and methods

### 2.1 Animals

All experimental procedures were approved by the Animal Ethics Committee of Oita University (Approval No. 235701). Male C57BL/6 mice were purchased from The Jackson Laboratory Japan (Kanagawa, Japan). Three-month-old mice were assigned to the young group (n = 12), whereas 18-month-old mice were assigned to the aged group (n = 12). Each age group was further divided into two subgroups based on whether the mice engaged in spontaneous physical activity using a running wheel (VWR) or remained in a standard housing without access to the running wheel (SED). Accordingly, the following four groups were established: Young_SED, Young_VWR, Aged_SED, and Aged_VWR (n = 6 per group). All animals were housed under controlled environmental conditions (25 °C, 12 h light/dark cycle) with *ad libitum* access to standard laboratory chow and water. Only male mice were used in this study to avoid variability caused by the estrous cycle, which affects the voluntary running behavior. Female mice tend to run longer distances and exhibit greater day-to-day variability than males (Manzanares et al., 2018). The use of male mice helped minimize confounding factors and ensured consistency in activity levels.

### 2.2 Voluntary wheel running protocol

The VWR protocol was implemented by housing mice for 8 weeks in individual cages (RWC-15; Melquest, Toyama, Japan) equipped with a running wheel (diameter, 140 mm), allowing unrestricted access to voluntary running. Running distances were recorded every 24 h from the first to the last day of the protocol. The SED group mice were housed in standard cages without a running wheel for the same duration. The activity levels of mice in the SED groups were not recorded.

### 2.3 Muscle sampling

After the 8-week housing period, body weight was measured, and the mice were euthanized by cervical dislocation within 1 h of the final VRW. The soleus (SOL; predominantly slow-twitch) and plantaris (PLAN; predominantly fast-twitch) muscles were excised from the posterior region of both the hindlimbs, and their wet weight was measured. Muscles harvested from the left side were rapidly frozen in isopentane for subsequent histological and immunohistochemical analyses and stored at −80 °C until further analysis. Muscles from the right side were immediately frozen in liquid nitrogen for real-time polymerase chain reaction (RT-PCR) analysis and stored at −80 °C until further analysis.

### 2.4 Histological and immunohistochemical analyses

Muscle samples from the left hindlimb were used for histological and immunohistochemical analyses. Frozen transverse sections (8 μm thick) of the SOL and PLAN were prepared at −25 °C using a cryostat (CM 1860; Leica Biosystems, Nussloch, Germany). The sections were fixed in 4% paraformaldehyde (PFA; Fujifilm Wako Pure Chemical Co.) prepared in phosphate-buffered saline (PBS) for 11 min, rinsed with PBS, and stained with hematoxylin and eosin (H&E). The stained sections were mounted in 90% glycerol. Images were captured using a light microscope (IX70; Olympus, Tokyo, Japan) with a ×20 objective lens (UPLFLN; Olympus). The ImageJ software (ver. 1.53; NIH, Bethesda, MD, United States) was used to measure the whole muscle cross-sectional area (CSA). From the muscle belly, 150 myofibers were randomly selected for measuring the myofiber CSA.

For immunohistochemical analysis, the sections were fixed in 4% PFA in PBS for 11 min and blocked with 4% Block Ace powder (KAC, Kyoto, Japan) for 60 min at room temperature (RT). The sections were incubated overnight at 4 °C with the following primary antibodies: rabbit anti-lymphatic endothelial hyaluronan receptor 1 (LYVE-1; 1:1000; 103-PA50AG; Relia Tech GmbH, Wolfenbüttel, Germany; RRID: AB_2876870) and rat anti-CD31 (1:50; 550274; BD Biosciences, San Jose, US-CA, CA; RRID: AB_393571). The sections were washed with PBS and further incubated in the dark for 60 min at RT with Alexa Fluor 488-conjugated goat anti-rabbit IgG (1:400; A-11008; Thermo Fisher Scientific, Waltham, US-MA; RRID: AB_143165) and Alexa Fluor 568-conjugated goat anti-rat IgG (1:400; A-11077; Thermo Fisher Scientific; RRID: AB_2534121). The stained sections were mounted in 90% glycerol. Fluorescent images were acquired using a fluorescence microscope (IX70; Olympus) with a ×20 objective lens (UPLFLN; Olympus). The contrast of images was adjusted using Adobe Photoshop (Adobe, San Jose, CA, United States). The ImageJ software (ver. 1.53; NIH, Bethesda, MD, United States) was used to count LYVE-1-positive structures as lymphatic vessels and CD31-positive structures as blood capillaries (Kivelä et al., 2007a). Data were normalized by area (density per unit area) and number of myofibers (ratio to myofibers). Five random areas, measuring 300 μm × 300 μm, were selected from the immunohistochemical staining images and used for analysis. Please refer to Supplementary Table S1 for the mean ± standard deviation values of lymphatic vessels, blood capillaries, and myofibers analyzed in each group.

To identify myofiber types, the sections were incubated overnight at 4 °C with primary antibodies, including rabbit anti-slow skeletal myosin heavy chain (Type I myofiber; 1:200; ab234431; Abcam, Waltham, MA; RRID: AB_3076242) and rat anti-laminin alpha-2 (laminin; 1:100; sc-59854; Santa Cruz Biotechnology; Dallas, TX; RRID: AB_784266). The sections were washed with PBS and further incubated in the dark for 60 min at RT with Alexa Fluor 488-conjugated goat anti-rat IgG (1:400; A-11006; Thermo Fisher Scientific; RRID: AB_2534074) and Alexa Fluor 568-conjugated goat anti-rabbit IgG (1:400; A-11011; Thermo Fisher Scientific; RRID: AB_143157). The stained sections were mounted in 90% glycerol and imaged with a fluorescence microscope using a ×10 objective lens (UPLFLN; Olympus). Myofibers were defined as cells surrounded by laminin-positive boundaries, whereas those positive for slow myosin heavy chain were identified as Type I myofibers. The ImageJ software was used to quantify the total number of myofibers, and the quantity of Type I myofibers relative to the total myofiber count was calculated for each section of the SOL and PLAN.

### 2.5 Real-time reverse transcription PCR

Total RNA was isolated from the right SOL and PLAN using Isogen II (Nippon Gene, Tokyo, Japan) in accordance with the manufacturer’s instructions. RNA concentration was measured using a DS-11 spectrophotometer (DeNovix, Wilmington, DE, United States). Complementary DNA (cDNA) was synthesized from total RNA using SuperScript VILO Master Mix (Thermo Fisher Scientific). Real-time PCR was performed using Fast SYBR Green Master Mix (Thermo Fisher Scientific) on a StepOnePlus Real-Time PCR System (Applied Biosystems). The relative mRNA expression levels of VEGF-A, VEGF-C, VEGF-D, VEGFR-2, VEGFR-3, tumor necrosis factor (TNF)-α, and interleukin (IL)-1β were quantified using the comparative Ct method (ΔΔCt) and normalized to glyceraldehyde-3-phosphate dehydrogenase (GAPDH) as an internal control. All primers were purchased from Greiner Bio-One (Kremsmünster, Austria). Primer sequences are listed in Supplementary Table S2.

### 2.6 Statistical analysis

All data are presented as mean ± standard deviation (SD). Two-sample *t*-tests were used to compare running distances for the Young_VWR and Aged_VWR groups. Two-way analysis of variance (ANOVA) was conducted to evaluate the effects of Age (Young vs. Aged), Run (SED vs. VWR), and their interaction. When a significant interaction was detected, one-way ANOVA followed by Tukey’s *post hoc* test was applied. For significant main effects without interaction, pairwise comparisons were conducted using the *t*-test. A p-value <0.05 was considered statistically significant. All statistical analyses were performed using Modified R Commander 4.0.2 (https://home.hirosaki-u.ac.jp/pteiki/r/).

## 3 Results

### 3.1 Running performance and physical parameters

Spontaneous running activity was compared between the Young_VWR and Aged_VWR groups. The total running distance over 8 weeks in the Young_VWR group was approximately twice as high (456.6 ± 93.1 km) as in the Aged_VWR group (221.0 ± 58.2 km) (p < 0.01).

At the end of the housing period, body mass was not significantly affected by VWR (p = 0.64); however, aged mice exhibited significantly higher body mass than young mice, regardless of the activity level. Both the Aged_SED (32.9 ± 1.2 g) and Aged_VWR (32.3 ± 1.3 g) groups exhibited significantly higher body mass than their respective Young groups (Young_SED: 26.7 ± 1.8 g; Young_VWR: 25.6 ± 0.8 g) (Table 1). Data on body mass changes over the 8-week intervention period in the VWR group are provided in Supplementary Table S3.

**TABLE 1:** Comparison of running performance and physical parameters in different groups.

Evaluation criteria	Young_SED	Young_VWR	Aged_SED	Aged_VWR	Interaction and main effects
Running distance(km)	―	456.6 ± 93.1	―	221.0 ± 58.2^††^	―
Body mass(g)	26.7 ± 1.8	25.6 ± 0.8	32.9 ± 1.2^**^	32.3 ± 1.3^††^	Int: p = 0.64 Age: p < 0.01 Run: p = 0.15
SOL mass(mg)	10.6 ± 0.9	11.4 ± 0.8	11.8 ± 0.6^*^	12.2 ± 1.0	Int: p = 0.60 Age: p < 0.05 Run: p = 0.12
PLAN mass(mg)	20.7 ± 0.8	21.2 ± 2.5	21.7 ± 1.5	22.1 ± 1.2	Int: p = 0.95 Age: p = 0.19 Run: p = 0.54

Int indicates interaction. Data are expressed as means ± SD. * <0.05 vs. Young_SED, group. ** <0.01 vs. Young_SED, group. †† <0.01 vs. Young_VWR, group.

The SOL mass was not significantly affected by VWR (p = 0.60). However, a significant main effect of age was detected (p < 0.05), with Aged_SED (11.8 ± 0.6 mg) and Aged_VWR (12.2 ± 1.0 mg) group mice showing greater muscle mass than the respective Young group mice (Young_SED: 10.6 ± 0.9 mg; Young_VWR: 11.4 ± 0.8 mg). For the PLAN mass, no significant interaction (p = 0.95) or main effect of Age (p = 0.19) and Run (p = 0.54) was detected. The values remained relatively comparable across the groups. Additionally, the mass of each muscle was normalized to the body weight (Supplementary Table S4).

### 3.2 Histological changes in muscle morphology

**FIGURE 1:**
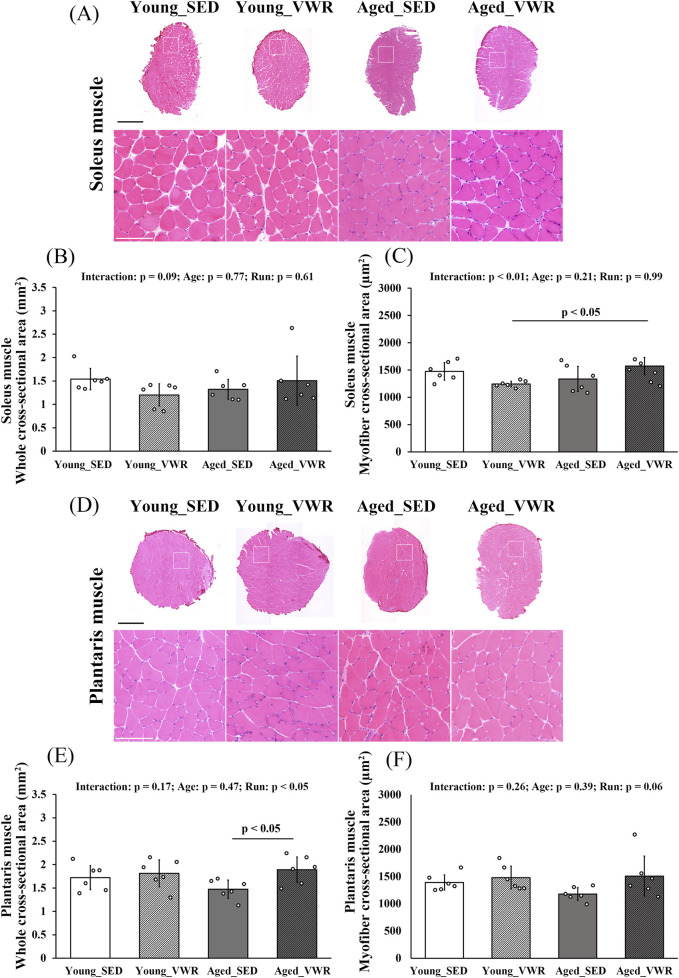
**(A)** Representative hematoxylin and eosin (H&E)-stained images of the soleus muscle. Enlarged views of the regions outlined by white boxes are also shown. **(B)** Quantification of the whole cross-sectional area of the soleus muscle. **(C)** Quantification of the myofiber cross-sectional area of the soleus muscle. **(D)** Representative H&E-stained images of the plantaris muscle. Enlarged views of the regions outlined by white boxes are also shown. **(E)** Quantification of the whole cross-sectional area of the plantaris muscle. **(F)** Quantification of the myofiber cross-sectional area of the plantaris muscle. Scale bars = 1 mm (full image) and 100 µm (enlarged image). Data are presented as means ± SD.

To assess morphological changes induced by VWR, we analyzed both whole muscle CSA and myofiber CSA. In the SOL (Figure 1A), whole muscle CSA showed no significant interaction between Age and Run (p = 0.09) and no main effects of Age (p = 0.77) and Run (p = 0.61) (Figure 1B). By contrast, myofiber CSA showed a significant interaction between Age and Run (p < 0.01); the Aged_VWR group (1572.0 ± 160.5 µm^2^) was significantly greater than the Young_VWR group (1242.8 ± 51.6 µm^2^), with no difference between Young_SED (1475.1 ± 161.9 µm^2^) and Aged_SED (1337.0 ± 229.1 µm^2^) (Figure 1C).

In the PLAN (Figure 1D), whole muscle CSA showed no significant Age × Run interaction (p = 0.17) but a significant main effect of Run (p < 0.05). Post hoc comparisons indicated that Old_VWR (1.89 ± 0.27 mm^2^) had greater whole muscle CSA than Old_SED (1.47 ± 0.19 mm^2^), whereas the Young groups did not differ (Figure 1E). For myofiber CSA, there was no significant Age × Run interaction (p = 0.26) and no main effects of Age (p = 0.39) or Run (p = 0.06) (Figure 1F).

### 3.3 Changes in myofiber type composition

**FIGURE 2:**
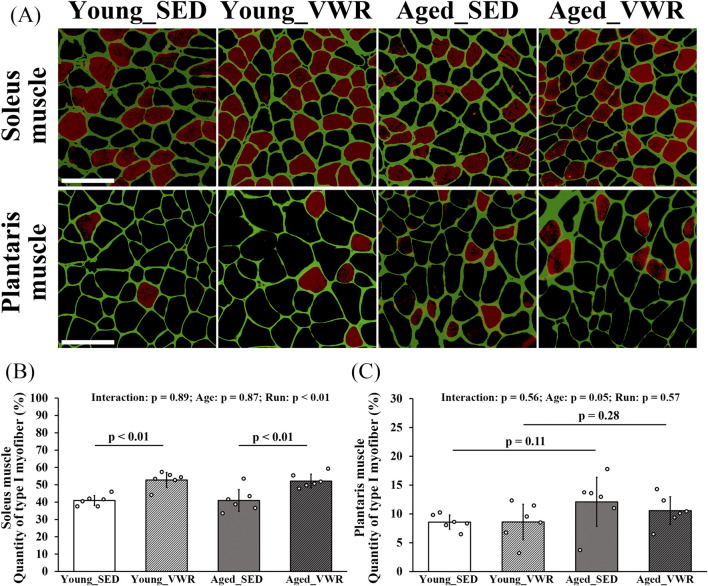
**(A)** Images of the soleus and plantaris muscle sections immunostained for laminin (green) and Type I myofibers (red). The quantity of Type I myofibers in the soleus **(B)** and plantaris muscle **(C)**. Scale bars = 100 μm. Data are presented as means ± SD.

The images of SOL and PLAN sections immunostained for Type I myofibers are presented in Figure 2A. The quantity of Type I myofibers in the SOL showed no significant interaction between Age and Run (p = 0.89); however, there was a significant main effect of Run (p < 0.01). Notably, both Young_VWR (52.8% ± 4.3%) and Aged_VWR (52.1% ± 4.0%) group mice showed significantly higher quantities of Type I myofibers in the SOL than Young_SED (41.0% ± 2.8%) and Aged_SED (40.9% ± 6.4%) group mice (Figure 2B). In the PLAN, the quantity of type I myofibers (two-factor model: Age, Run) showed no significant Age × Run interaction (p = 0.56). Using a two-sided α = 0.05 (significant if p < 0.05), the main effect of Age was borderline (p = 0.05) and was not considered significant; *post hoc* comparisons likewise showed no differences between young and aged mice within either activity condition (Young_SED vs. Aged_SED: p = 0.11; Young_VWR vs. Aged_VWR: p = 0.28) (Figure 2C). Data on the total number of myofibers and the number of Type I myofibers in each muscle are provided in Supplementary Table S5.

### 3.4 Changes in the lymphatic vessels and blood capillaries

**FIGURE 3:**
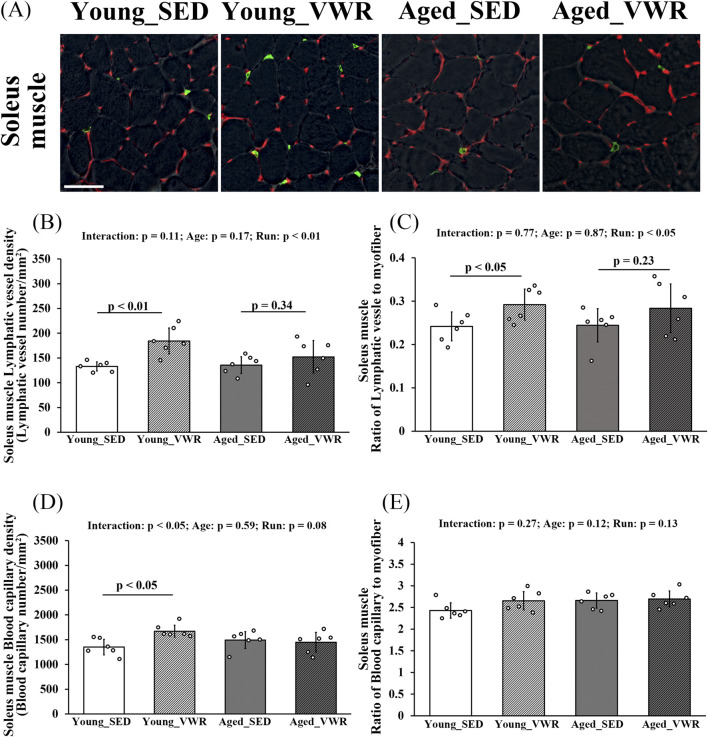
**(A)** Immunostaining images of the soleus muscles (gray: myofiber imaged via phase-contrast microscopy; green: LYVE-1-positive lymphatic vessels; red: CD31-positive blood capillaries). Quantification of lymphatic vessels density **(B)** and the ratio of lymphatic vessels to myofibers **(C)**. Quantification of blood capillary density **(D)** and the ratio of blood capillaries to myofibers **(E)**. Scale bars = 50 μm. Data are presented as means ± SD.

To quantify lymphatic vessel density and the ratio of lymphatic vessels to myofibers in SOL, we immunostained the muscle sections for LYVE-1 and CD31 (Figure 3A). In the SOL, no significant interaction between Age and Run was observed for lymphatic vessel density (LVD) (p = 0.11), but a significant main effect of Run was detected in Young_SED vs. Young_VWR or Aged_SED vs. Aged_VWR (p < 0.01). The LVD in Young_VWR group mice (184.4 ± 26.1/mm^2^) was significantly higher than that in Young_SED group mice (133.0 ± 9.4/mm^2^), whereas no significant difference was found between the Aged groups (Aged_SED: 135.6 ± 17.0/mm^2^, Aged_VWR: 152.2 ± 33.1/mm^2^) (Figure 3B). The ratio of lymphatic vessels to myofibers showed no significant interaction between Age and Run (p = 0.77); however, a significant main effect of Run was detected in Young_SED vs. Young_VWR or Aged_SED vs. Aged_VWR (p < 0.05). The ratio of lymphatic vessels to myofibers in the Young_VWR group (0.29 ± 0.04) was significantly higher than that in the Young_SED group (0.24 ± 0.03), whereas no significant difference was observed between the aged groups (Aged_SED: 0.24 ± 0.04, Aged_VWR: 0.28 ± 0.06) (Figure 3C).

For blood capillary density in the SOL, a significant Age × Run interaction was detected (p < 0.05): mice in the Young_VWR group showed higher capillary density (1670.4 ± 121.6/mm2) than Young_SED (1351.5 ± 157.2/mm2), whereas no difference was observed between Aged_SED (1493.0 ± 169.3/mm2) and Aged_VWR (1448.5 ± 198.3/mm2) (Figure 3D). The ratio of blood capillaries to myofibers showed no significant Age × Run interaction (p = 0.27), and there were no main effects of Age (p = 0.12) or Run (p = 0.13) (Figure 3E).

**FIGURE 4:**
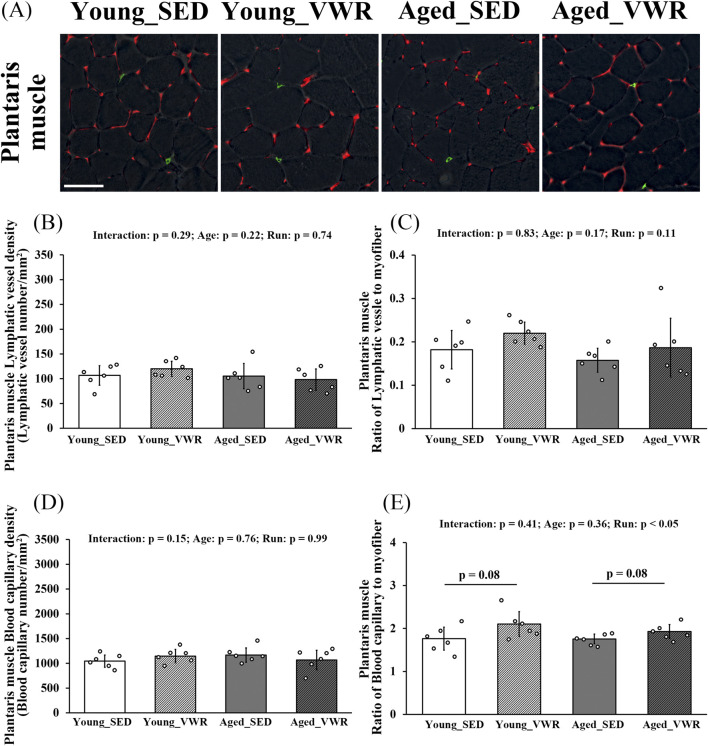
**(A)** Immunostaining images of the plantaris muscles (gray: myofiber imaged via phase-contrast microscopy; green: LYVE-1-positive lymphatic capillaries; red: CD31-positive blood capillaries). Quantification of lymphatic vessels density **(B)** and the ratio of lymphatic vessels to myofibers **(C)**. Quantification of blood capillary density **(D)** and the ratio of blood capillaries to myofibers **(E)**. Scale bars = 50 μm. Data are presented as means ± SD.

To determine the density and myofiber-normalized ratios of lymphatic vessels and blood capillaries in the PLAN, we immunostained muscle sections for LYVE-1 and CD31 (Figure 4A). In the PLAN, LVD showed no significant Age × Run interaction (p = 0.29) and no main effects of Age (p = 0.22) or Run (p = 0.74) (Figure 4B). The ratio of lymphatic vessels to myofibers likewise showed no significant Age × Run interaction (p = 0.83) and no main effects of Age (p = 0.17) or Run (p = 0.11) (Figure 4C).

For blood capillary density in the PLAN, there was no significant Age × Run interaction (p = 0.15) and no main effects of Age (p = 0.76) or Run (p = 0.99) (Figure 4D). The ratio of blood capillaries to myofibers showed no significant Age × Run interaction (p = 0.41). There was a main effect of Age (p = 0.05); however, *post hoc* comparisons revealed no differences between SED and VWR within age (Young_SED vs. Young_VWR: p = 0.08; Aged_SED vs. Aged_VWR: p = 0.08) (Figure 4E).

### 3.5 Changes in mRNA expression of VEGF family members

**FIGURE 5:**
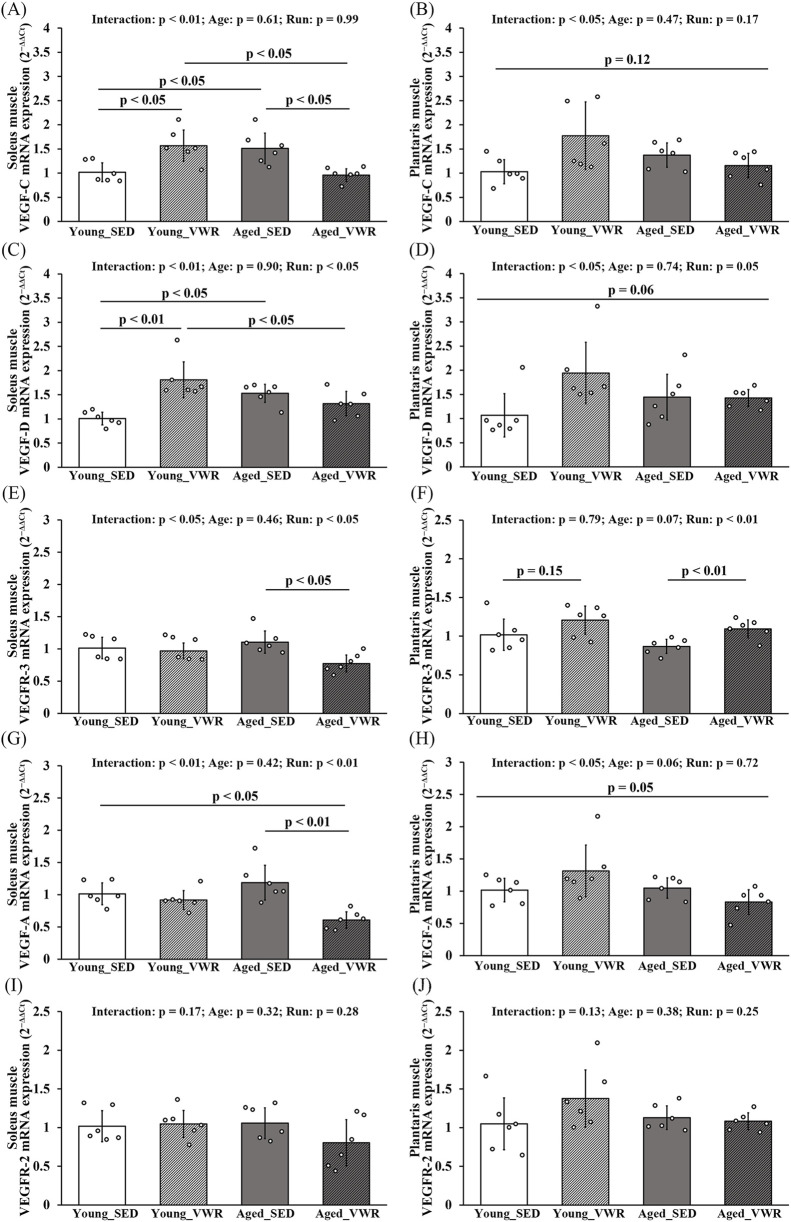
mRNA expression levels of VEGF family members in the soleus **(A,C,E,G,I)** and plantaris **(B,D,F,H,J)** muscles. mRNA levels of the lymphangiogenic factors VEGF-C and VEGF-D, and their receptor VEGFR-3 **(A–F)**. mRNA levels of the angiogenic factor VEGF-A and its receptor VEGFR-2 **(G–J)**. Data are presented as means ± SD.

In the SOL, VEGF-C expression showed a significant Age × Run interaction (p < 0.01): levels were higher in the Young_VWR and Aged_SED groups than in Young_SED, whereas the Aged_VWR group exhibited lower levels than both Young_VWR and Aged_SED (Figure 5A). In the PLAN, VEGF-C also showed a significant Age × Run interaction (p < 0.05), but *post hoc* comparisons did not reach significance (p = 0.12) (Figure 5B). The expression of VEGF-D in the SOL paralleled VEGF-C, except that no significant difference was found between Young_VWR and Aged_VWR (Figure 5C). In the PLAN, VEGF-D again showed a significant Age × Run interaction (p < 0.05), but group differences did not reach significance (p = 0.06) (Figure 5D). VEGFR-3 expression showed a significant Age × Run interaction in the SOL (p < 0.05), with Aged_VWR lower than the other groups (Figure 5E). In the PLAN, no Age × Run interaction was observed (p = 0.41), but a main effect of Run was detected (p < 0.05); *post hoc* comparisons indicated higher expression in Aged_VWR than Aged_SED (Figure 5F).

VEGF-A expression in the SOL exhibited a significant Age × Run interaction (p < 0.01), with Aged_VWR lower than Young_SED and Aged_SED (Figure 5G). In the PLAN, an Age × Run interaction was also detected (p < 0.05), although pairwise differences were marginal and did not reach significance (p = 0.05) (Figure 5H). VEGFR-2 expression showed no Age × Run interaction and no main effects of Age or Run in either the SOL or PLAN (Figures 5I,J).

### 3.6 Changes in mRNA expression of inflammatory cytokines

**FIGURE 6:**
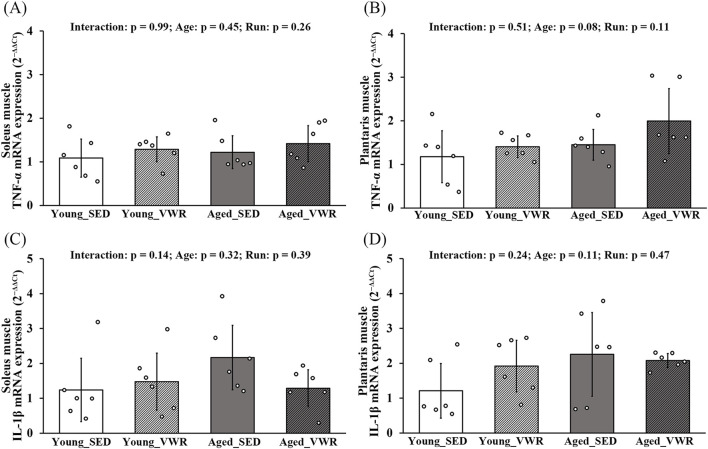
mRNA expression levels of TNF-α **(A,B)** and IL-1β **(C,D)** in the soleus and plantaris muscles. Data are presented as means ± SD.

In both SOL and PLAN muscles, TNF-α expression showed no significant Age × Run interaction and no main effects of Age or Run (Figures 6A,B). Similarly, IL-1β expression showed no Age × Run interaction and no main effects of Age or Run in either muscle (Figures 6C,D).

## 4 Discussion

### 4.1 Morphological adaptations to VWR and aging

In this study, we investigated the effects of 8 weeks of VWR on histological changes in intramuscular lymphatic vessels and blood capillaries, using both young and aged mice. We focused on the SOL, a muscle rich in slow-twitch myofibers, and the PLAN, which is predominantly composed of fast-twitch myofibers (Bloemberg and Quadrilatero, 2012; Mobley et al., 2017).

No significant differences in whole muscle CSA or myofiber CSA were observed between the Young_SED and Aged_SED groups for both SOL and PLAN. However, in Aged_VWR, myofiber CSA was significantly larger for SOL than for the Young_VWR group. Previous study have shown that VWR reduces the myofiber CSA in young mice (Pellegrino et al., 2005). In contrast, VWR has been reported to increase myofiber CSA in aged mice (Baek et al., 2022). The results of this study indicate morphological adaptation due to changes in VWR responsiveness with aging. However, because muscle function, such as muscle strength or fatigue resistance, was not directly evaluated in this study, this interpretation should be considered preliminary and warrants further investigation. In young mice, VWR induced an increase in Type I myofibers in the SOL. VWR upregulates Type I myosin isoforms while downregulating Type IIa isoform in the SOL (Fuller et al., 2006; Hyatt et al., 2019); our findings are consistent with these observations. In contrast, no significant changes in myofiber-type composition were observed in the PLAN of young mice. VWR does not induce a shift from Type II to Type I myofibers in the PLAN (Waters et al., 2004), which is indicative of a limited response of the PLAN to endurance-based stimuli such as VWR.

### 4.2 Vascular remodeling in SOL induced by VWR

In the SOL of young mice, VWR significantly increased the LVD, the ratio of lymphatic vessels to myofibers. Lymphangiogenesis is primarily driven by the binding of VEGF-C and VEGF-D to their receptor VEGFR-3 (Tammela and Alitalo, 2010; Ji, 2012; Suarez et al., 2023). In our study, the expression of VEGF-C and VEGF-D was significantly increased by VWR, corresponding with the observed increase in LVD and the ratio of lymphatic vessels to myofibers. These results indicate that VEGF-C and VEGF-D may contribute to the VWR-induced increase in lymphangiogenesis. However, the VEGFR-3 mRNA levels did not change despite VWR. Kawashima et al. reported that the expression of VEGF-C and VEGF-D was decreased in muscle atrophy, whereas that of VEGFR-3 remained stable (Kawashima et al., 2021). Accordingly, the expression of these factors in skeletal muscle may not always be tightly correlated.

In contrast to our findings with VWR, previous treadmill-based exercise studies have not demonstrated lymphangiogenic responses in skeletal muscle (Kivelä et al., 2007b; Greiwe et al., 2016). Compared with VWR, the total daily running distance achieved with treadmill-based exercise is generally shorter. Moreover, treadmill-based exercise involves higher exercise intensity and exercise within a limited time frame. It is possible that differences in these exercise modalities may influence the development of intramuscular lymphatic vessels.

The increase in intramuscular blood capillary density in the SOL in response to VWR has also been documented (Hyatt et al., 2019), and our study corroborates these findings. While angiogenesis is mediated by the binding of VEGF-A to its receptor VEGFR-2 (Kudelka et al., 2013; Wang et al., 2020; Shah et al., 2025), no significant changes in VEGF-A or VEGFR-2 mRNA levels were observed in the SOL of young mice following VWR in this study. Prior research, including embryological studies and *in vitro* experiments, suggests that blood vessel formation precedes lymphangiogenesis (Martinez-Corral et al., 2015; Osaki et al., 2018; Hu et al., 2024; Schoofs and Mäkinen, 2024), and angiogenesis in skeletal muscles reportedly occurs earlier than lymphangiogenesis (Kawashima et al., 2021; Pu et al., 2021). Therefore, in our study, it is possible that at the time of muscle harvest, angiogenesis had already been completed and VEGF-A expression was no longer required. However, the reason for the unchanged VEGF-A expression remains unclear and warrants further investigation, including consideration of the timing of muscle sampling. Notably, VEGF-D has also been implicated in angiogenesis in skeletal muscles (Rissanen et al., 2003; Kärkkäinen et al., 2009). In our study, increased VEGF-D expression in the SOL of the Young_VWR group coincided with greater blood capillary density, suggesting a possible involvement of VEGF-D in capillary formation.

In contrast, no significant differences were observed in the blood capillary to myofiber ratio in the SOL among groups. This may be attributable to the evaluation being based on the numbers of myofibers, lymphatic vessels, and blood capillaries per unit area. Specifically, in the Young_VWR group, the slightly smaller myofiber CSA (Figure 1C) likely resulted in a greater number of myofibers within the analyzed regions, thereby influencing the ratio (Supplementary Table S1). Moreover, the quantity of type I myofibers increased in the VWR groups, suggesting that the observed changes in myofiber CSA may reflect physiological adaptations induced by exercise. However, further studies are needed to clarify these mechanisms in detail.

VWR increased the proportion of type I myofibers in the SOL, which are generally considered fatigue-resistant (Carrell et al., 2016). Type I myofibers possess high oxidative capacity (Xiao et al., 2021) and require a sustained supply of oxygen to maintain prolonged contractile activity. This continuous oxygen demand may have contributed to the observed increase in capillary density. The resulting increase in interstitial fluid leakage from blood capillaries could in turn enhance lymphatic vessel formation, which plays a role in fluid clearance (Angeli and Lim, 2023; Hu et al., 2024). In contrast, no changes in lymphangiogenesis or angiogenesis were observed in the PLAN. This muscle did not undergo a shift toward a slow-twitch phenotype following VWR, which provides a plausible explanation for the absence of vascular adaptations in this muscle.

It should be noted, however, that in mouse skeletal muscle, type IIa myofibers are known to exhibit the highest oxidative capacity (Gouspillou et al., 2014; Thomas et al., 2014), and this factor should be taken into account when interpreting the present findings. Therefore, a more comprehensive evaluation of myofiber type composition, including type IIa myofibers, together with functional assessments of oxidative capacity such as succinate dehydrogenase (SDH) activity, remains an important subject for future studies.

### 4.3 Age-related differences in vascular responsiveness

Although aging has been associated with a decline in the number of lymphatic vessels in dermal, heart, and rat skeletal muscles (Karaman et al., 2015; Taketa et al., 2024; Roh et al., 2025), we observed no significant differences in LVD between young and aged mice. This might be due to the lack of age-related changes in myofiber-type composition in our samples. Previous reports have indicated a decrease in the expression of lymphangiogenic factor with aging (Taketa et al., 2024); however, in our study, the expression levels of these factors were significantly increased in the SOL of aged mice. This may reflect a compensatory upregulation in response to an age-related decline in the lymphatic vasculature.

VEGF-C and VEGF-D are induced by proinflammatory cytokines, such as TNF-α and IL-1 (Zhang et al., 2007; Ji, 2012; Schwager and Detmar, 2019). Kawanishi and Machida (2021) reported elevated levels of these cytokines in the skeletal muscle of aged mice (27-month-old), which are near the end of their lifespan. In contrast, in the present study, mRNA levels of TNF-α and IL-1 did not differ significantly between young and aged mice, suggesting that the upregulation of VEGF-C and VEGF-D was not driven by proinflammatory cytokines. We selected 18-month-old mice, which are considered to retain relatively high activity levels (Shoji and Miyakawa, 2019). This selection was intended to evaluate whether VWR could help maintain physical activity and induce changes in intramuscular lymphatic vessels in aged mice. Notably, small differences in age among aged mice can lead to significant alterations in gene expression profiles (Kang et al., 2022). The discrepancy between our findings and those of Kawanishi and Machida (2021) may be attributable to differences in the age of the mice used.

Therapeutic strategies targeting VEGF-C or VEGF-D signaling may help overcome the impaired vascular responsiveness in aged skeletal muscle. Anisimov et al. (2009) reported that sustained overexpression of activated VEGF-C and VEGF-D in skeletal muscle using recombinant adeno-associated virus vectors significantly increased the densities of both blood and lymphatic vessels, improved tissue perfusion, and enhanced lymphatic drainage. These results implied that exogenous delivery of VEGF-C or VEGF-D could potentially restore lymphangiogenesis and might mitigate aging-associated muscle decline. Despite an increase in Type I myofibers following VWR, aged mice did not show corresponding increases in LVD and blood capillary density in the SOL. This discrepancy may be attributable to differences in the expression patterns of lymphangiogenic factors between young and aged mice. Blood capillary responsiveness to exercise is known to decline with age (Hendrickse et al., 2021), and a similar phenomenon may explain the lack of capillary adaptation observed in our aged cohort. Furthermore, age-related functional decline in lymphatic vessels has also been reported (Ji, 2018; Yang et al., 2023), which is suggestive of reduced responsiveness to exercise-induced stimuli. Because other molecules, such as angiopoietins and platelet-derived growth factors, are also critically involved in lymphangiogenesis (Cao et al., 2004; Korhonen et al., 2022; Hu et al., 2024), future studies should investigate their contributions and interactions in this context. In particular, the significantly shorter total running distance observed for aged mice compared with that for young mice over the 8-week VWR period may reflect an underlying decline in vascular and lymphatic function. Clarifying the relationship between vessel density and endurance performance will be essential to validate this hypothesis in future studies.

### 4.4 Methodological limitations and future studies

LYVE-1 is commonly used as a marker for lymphatic vessel structures; however, it can also be expressed by macrophages, particularly under inflammatory conditions (Hall et al., 2012; Zhang et al., 2021). On the other hand, voluntary wheel running (VWR) is reported to be a non-damaging form of exercise. For example, even in Duchenne muscular dystrophy model mice, VWR does not induce muscle injury (Monceau et al., 2022). In this study, no significant differences in the mRNA expression levels of the pro-inflammatory cytokines TNF-α and IL-1β were observed among groups, suggesting that inflammation was unlikely to have occurred. Nevertheless, protein-level analyses were not performed, and this remains an important limitation and direction for future studies. Moreover, the LYVE-1–positive cells identified in this study colocalized with VEGFR-3 (Supplementary Figure S1), another well-established marker of lymphatic vessels (Weber et al., 2018). Therefore, it is considered appropriate to regard LYVE-1–positive cells as lymphatic vessels in the present study.

In addition, our study was focused primarily on histological changes in lymphatic vessels and did not address their physiological functions, which represents a key limitation. Future studies using functional inhibition or stimulation approaches will be essential to elucidate the physiological roles of intramuscular lymphatic vessels and their contribution to exercise-induced muscle adaptation.

Furthermore, another significant limitation is that our analysis was confined to two-dimensional techniques such as immunohistochemical staining. With respect to blood capillaries in skeletal muscle, previous studies employing three-dimensional technologies such as confocal microscopy and two-photon laser scanning microscopy have demonstrated more comprehensive insights (Umek et al., 2019; Shimotsu et al., 2021). However, three-dimensional imaging of intramuscular lymphatic vessels has not yet been established and represents an important challenge for future studies. Once such techniques are developed, they may enable more precise analyses of exercise- or disease-induced alterations in the distribution and morphology of intramuscular lymphatic vessels. Moreover, they are expected to facilitate the evaluation of lymphatic fluid retention capacity using macromolecular tracer techniques, thereby contributing to more functional assessments.

As a side note, the lack of pre-intervention weight measurements in the SED group, coupled with the relatively small sample size (n = 6 per group), which should be considered when interpreting the results.

### 4.5 Conclusion

In conclusion, this study revealed that 8 weeks of VWR significantly increased LVD and blood capillary density in the SOL of young mice, possibly via VEGF-C and VEGF-D upregulation. These changes appear to represent an adaptive response to the increased oxygen demand associated with a VWR-induced shift toward a slow-twitch phenotype. In contrast, no changes in LVD and blood capillary density were observed in aged mice, possibly due to differences in their responsiveness to exercise stimulus.

## Data Availability

The original contributions presented in the study are included in the article/Supplementary Material, further inquiries can be directed to the corresponding author.
