# Supplementary material for: Voluntary wheel running promotes lymphangiogenesis in slow-twitch muscle in young mice
Source: Front Physiol. 2025 Oct 10;16:1654445. doi: 10.3389/fphys.2025.1654445 (PMC12549571; doi:10.3389/fphys.2025.1654445)
Supplement: Supplementary file 1 [file DataSheet4.docx]

|  | **Week 1** | **Week 2** | **Week 3** | **Week 4** | **Week 5** | **Week 6** | **Week 7** | **Week 8** |
| --- | --- | --- | --- | --- | --- | --- | --- | --- |
| **Youn_VWR** | **24.2 ± 0.8** | **23.5 ± 0.8** | **23.8 ± 08** | **24.1 ± 0.9** | **24.0 ± 0.8** | **24.3 ± 1.2** | **24.9 ± 0.9** | **25.6 ± 0.8** |
| **Aged_VWR** | **34.4 ± 1.3** | **33.0 ± 1.4** | **32.8 ± 1.2** | **32.7 ± 1.3** | **32.8 ± 1.2** | **32.5 ± 1.2** | **32.7 ± 1.1** | **32.3 ± 1.3** |

**Supplemental Table S2. Changes in body mass in the VWR group during the 8-week intervention period (g)**
