# Supplementary material for: Voluntary wheel running promotes lymphangiogenesis in slow-twitch muscle in young mice
Source: Front Physiol. 2025 Oct 10;16:1654445. doi: 10.3389/fphys.2025.1654445 (PMC12549571; doi:10.3389/fphys.2025.1654445)
Supplement: Supplementary file 2 [file DataSheet6.docx]

| **Supplemental Table S3 Number of All myofibers and TypeⅠ myofibers** | | | | |
| --- | --- | --- | --- | --- |
|  | **Young_SED** | **Young_VER** | **Aged_SED** | **Aged_VWR** |
| **SOL**  **(Total myofibers)** | **849.3 ± 62.4** | **807.0 ± 124.1** | **787.3 ± 112.5** | **655.8 ± 98.6** |
| **SOL**  **(TypeⅠ myofibers)** | **347.3 ± 27.8** | **422.3 ± 53.5** | **323.8 ± 71.1** | **340.0 ± 44.3^†^** |
| **PLAN**  **(Total myofibers)** | **943.3 ± 166.1** | **973.0 ± 142.7** | **856.0 ± 76.5** | **781.8 ± 186.4** |
| **PLAN**  **(TypeⅠ myofibers)** | **80.3 ± 15.7** | **83.8 ± 36.1** | **101.8 ± 36.0** | **81.8 ± 23.2** |

Dates are expressed as means ± SD.
