# Supplementary material for: Voluntary wheel running promotes lymphangiogenesis in slow-twitch muscle in young mice
Source: Front Physiol. 2025 Oct 10;16:1654445. doi: 10.3389/fphys.2025.1654445 (PMC12549571; doi:10.3389/fphys.2025.1654445)
Supplement: Supplementary file 3 [file DataSheet3.docx]

| **Supplemental Table S1 Primers for real-time RT–PCR** | |
| --- | --- |
| **Gene** | **SYBR Green Primer (5′-3′)** |
| **VEGF-C** | Fwd: GTAAAAACAAACTTTTCCCTAATTC |
|  | Rev: TTTAAGGAAGCACTTCTGTGTGT |
| **VEGF-D** | Fwd: GCAAGACGAGACTCCACTGC |
|  | Rev: GGTGCTGAATGAGATCTCCC |
| **VEGFR-3** | Fwd: GCAGGAGGAGGAAGAGGAGC |
|  | Rev: TGCATGCTGGGTGGACTATCA |
| **VEGF-A** | Fwd: AGTGGCTTACCCTTCCTZTCTT |
|  | Rev: CGGGTCCTGCCCCATT |
| **VEGFR-2** | Fwd: TGTCAAGTGGCGGTAAAGG |
|  | Rev: CACAAAGCTAAAATACTGAGGACTTG |
| **TNF-α** | Fwd: CCTCCCTCTCATCAGTTCTA |
|  | Rev: ACTTGGTGGTTTGCTACGAC |
| **IL-1β** | Fwd: TCCAGGATGAGGACATGAGCAC |
|  | Rev: GAACGTCACACACCAGCAGGTTA |
| **GAPDH** | Fwd: GACGGCCGCATCTTCTTGTG |
|  | Rev: CTTCCCATTCTCGGCCTTGACTGT |
