# Supplementary material for: Voluntary wheel running promotes lymphangiogenesis in slow-twitch muscle in young mice
Source: Front Physiol. 2025 Oct 10;16:1654445. doi: 10.3389/fphys.2025.1654445 (PMC12549571; doi:10.3389/fphys.2025.1654445)
Supplement: Supplementary file 4 [file DataSheet5.docx]

| **Supplemental Table S2 Muscle Mass Normalized to Body Weight** | | | | | |
| --- | --- | --- | --- | --- | --- |
|  | **Young_SED** | **Young_VER** | **Aged_SED** | **Aged_VWR** | **Interaction**  **and**  **main effects** |
| **SOL mass (mg/g)** | **0.40 ± 0.05** | **0.44 ± 0.02** | **0.36 ± 0.03** | **0.38 ± 0.03^††^** | **Int: p = 0.38 Age: p < 0.01**  **Run: p < 0.05** |
| **PLAN mass (mg/g)** | **0.78 ± 0.06** | **0.83 ± 0.07** | **0.66 ± 0.05^**^** | **0.66 ± 0.05^††^** | **Int: p = 0.62 Age: p < 0.01**  **Run: p = 0.16** |

Int indicates interaction. Dates are expressed as means ± SD. ** < 0.01 vs Young_SED group. †† < 0.01 vs Young_VWR group.
