# Supplementary material for: Voluntary wheel running promotes lymphangiogenesis in slow-twitch muscle in young mice
Source: Front Physiol. 2025 Oct 10;16:1654445. doi: 10.3389/fphys.2025.1654445 (PMC12549571; doi:10.3389/fphys.2025.1654445)
Supplement: Supplementary file 5 [file DataSheet2.docx]

| **Supplemental Table S1. Number of myofibers, lymphatic vessels and blood capillaries in SOL and PLAN muscle** | | | | | |
| --- | --- | --- | --- | --- | --- |
|  |  | **Young_SED** | **Young_VWR** | **Aged_SED** | **Aged_VWR** |
| **SOL** | **Number of myofibers** | 252.0 ± 38.4 | 284.1 ± 22.1 | 253.5 ± 32.9 | 241.1 ± 23.2 |
|  | **Number of lymphatic vessels** | 59.8 ± 4.2 | 83.0 ± 11.7 | 61.0 ± 7.6 | 68.5 ± 14.9 |
|  | **Number of blood capillaries** | 608.2 ± 70.7 | 751.7 ± 54.7 | 671.8 ± 76.2 | 651.8 ± 89.2 |
| **PLAN** | **Number of myofibers** | 268.7 ± 25.2 | 247.8 ± 31.4 | 298.5 ± 26.0 | 250.8 ± 50.8 |
|  | **Number of lymphatic vessels** | 48.0 ± 8.9 | 54.0 ± 6.8 | 47.3 ± 11.5 | 44.2 ± 9.6 |
|  | **Number of blood capillaries** | 470.0 ± 55.8 | 515.8 ± 60.6 | 525.0 ± 65.2 | 479.7 ± 89.0 |
