# Supplementary material for: Voluntary wheel running promotes lymphangiogenesis in slow-twitch muscle in young mice
Source: Front Physiol. 2025 Oct 10;16:1654445. doi: 10.3389/fphys.2025.1654445 (PMC12549571; doi:10.3389/fphys.2025.1654445)
Supplement: Supplementary file 6 [file DataSheet1.docx]

Supplementary Material

# Supplementary Method:

**Analysis of the Colocalization of LYVE-1 and VEGFR-3**

To identify lymphatic vessels, frozen transverse sections of the soleus and plantaris muscles (8  µm thick) were fixed in 4% paraformaldehyde prepared in phosphate-buffered saline (PBS) for 10 min at room temperature (RT), followed by PBS washes. The sections were incubated overnight at 4 °C with the following primary antibodies: rabbit anti-LYVE-1 (1:1000; 103-PA50AG; Relia Tech GmbH, Wolfenbüttel, Germany; RRID: AB_2876870) and goat anti-VEGFR-3 (1:100; AF743; R&D Systems, Minneapolis, MN; RRID: AB_355563). After washing with PBS, the sections were incubated for 60 min at RT in the dark with the following secondary antibodies: Alexa Fluor 488-conjugated donkey anti-rabbit IgG (1:400; A-21206; Thermo Fisher Scientific; RRID: AB_2535792) and Alexa Fluor 568-conjugated donkey anti-goat IgG (1:400; A-11058; Thermo Fisher Scientific; RRID: AB_2534104). The stained sections were mounted in 90% glycerol and imaged using a fluorescence microscope with a 40× objective lens. Structures positive for both LYVE-1 and VEGFR-3 were identified as lymphatic vessels.


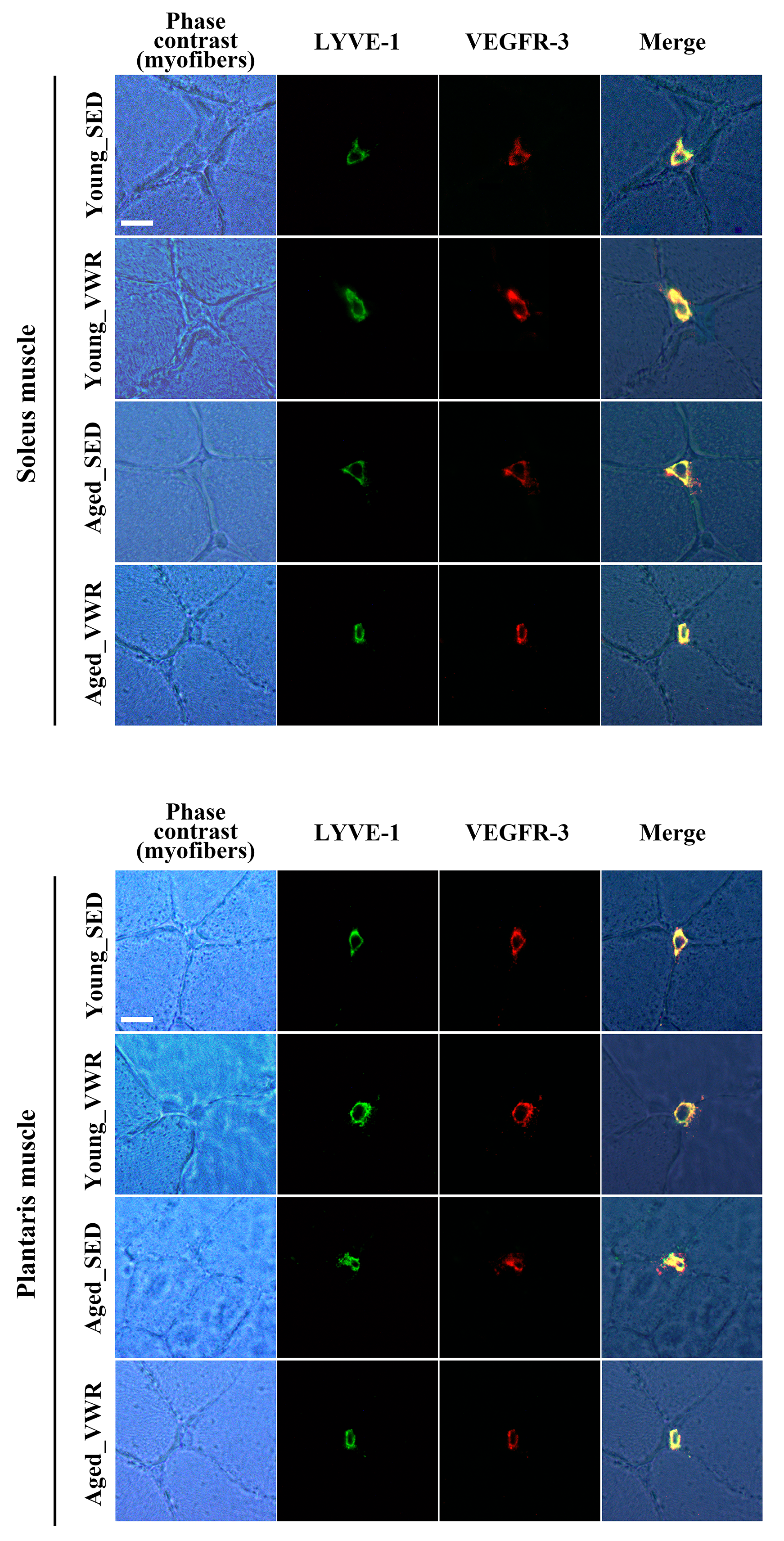


**Supplemental Figure S1. Coimmunofluorescence of LYVE-1 and VEGFR-3 in the soleus and plantaris muscles.**

Representative immunofluorescence images of the soleus and plantaris muscles. Muscle fibers are shown in gray (phase-contrast microscopy), LYVE-1-positive lymphatic vessels in green, and VEGFR-3-positive lymphatic vessels in red. Scale bars = 10 µm
